# Advances in Renal Cell Carcinoma Drug Resistance Models

**DOI:** 10.3389/fonc.2022.870396

**Published:** 2022-05-10

**Authors:** Yien Xiang, Ge Zheng, Jianfeng Zhong, Jiyao Sheng, Hanjiao Qin

**Affiliations:** ^1^ Department of Hepatobiliary and Pancreatic Surgery, Second Hospital of Jilin University, Changchun, China; ^2^ Department of Clinical Laboratory, Second Hospital of Jilin University, Changchun, China; ^3^ Department of Radiotherapy, The Second Hospital of Jilin University, Changchun, China

**Keywords:** renal cell carcinoma, *in vitro* model, *in vivo* model, drug resistance, PDX (patient derived xenograft)

## Abstract

Renal cell carcinoma (RCC) is the most common form of kidney cancer. Systemic therapy is the preferred method to eliminate residual cancer cells after surgery and prolong the survival of patients with inoperable RCC. A variety of molecular targeted and immunological therapies have been developed to improve the survival rate and prognosis of RCC patients based on their chemotherapy-resistant properties. However, owing to tumor heterogeneity and drug resistance, targeted and immunological therapies lack complete and durable anti-tumor responses; therefore, understanding the mechanisms of systemic therapy resistance and improving clinical curative effects in the treatment of RCC remain challenging. *In vitro* models with traditional RCC cell lines or primary cell culture, as well as *in vivo* models with cell or patient-derived xenografts, are used to explore the drug resistance mechanisms of RCC and screen new targeted therapeutic drugs. Here, we review the established methods and applications of *in vivo* and *in vitro* RCC drug resistance models, with the aim of improving our understanding of its resistance mechanisms, increasing the efficacy of combination medications, and providing a theoretical foundation for the development and application of new drugs, drug screening, and treatment guidelines for RCC patients.

## Introduction

Renal cell carcinoma (RCC) is the most prevalent type of renal malignancy, accounting for 90-95% of all renal cancers (1). Clear cell, papillary, and chromophobe are the most common types of RCC (2), while collecting duct carcinoma, renal medullary carcinoma, mucinous tubular and spindle cell carcinoma, papillary adenoma, and other pathological types are less common (3). More than 350,000 people worldwide are diagnosed with RCC, and approximately 140,000 people die annually (4). Approximately 30% of RCC patients have metastases at diagnosis and 30-70% of the tumors may relapse after surgery (5). Despite the promise of targeted therapies such as tyrosine kinase inhibitors (TKIs) and mTOR inhibitors, as well as immunotherapies like VEGF monoclonal antibodies and immune checkpoint inhibitors, on the extension of progression-free survival (PFS) and overall survival (OS) in patients with progressed or metastasized RCC, the patients eventually succumb to inevitable drug resistance. Recently, a number of studies have focused on the sophisticated mechanisms of drug resistance in RCC with the help of various drug resistance models. We reviewed original articles concerning drug resistance of RCCs that were published in the last 10 years. The drug resistance models used are summarized, with some key drug resistance mechanisms which were found by using the drug resistance models introduced in detail.

## 1 Traditional *In Vitro* and *In Vivo* Drug Resistance Models

### 1.1 Establishment Methods of Traditional Drug Resistance Models

As the most commonly used method, traditional drug resistance models have been established using commercial RCC cell lines. For *in vitro* models, parental RCC cells are exposed to the drugs either with a constant high concentration or gradually increasing concentrations for 20-30 generations or 3-6 months to acquire drug-specific resistance. There are two major ways to establish *in vivo* drug resistance in mouse models. Drug-resistant RCC cells can be directly implanted into nude mice subcutaneously or orthotopically. Drug-resistant mice could also be established *via* an initial subcutaneous or orthotopic injection of parental RCC cells and subsequent long-term oral feeding of the drugs. **Table 1** lists the studies on RCC drug resistance using traditional *in vitro* and *in vivo* models. Briefly, 786-O, A498, ACHN, and CAKI-1 are the four most commonly used RCC cell lines for the establishment of traditional *in vitro* and *in vivo* models. The TKIs sunitinib and sorafenib and the mTOR inhibitors temsirolimus and everolimus are major research objects. Furthermore, resistance to a single drug may be associated with many signaling pathways. A specific drug that could reverse the above signaling could be used as an alternative or combined treatment.

**Table 1:** Researches on RCC drug resistance using traditional *in-vitro* and *in-vivo* models.

Year	Model	Primary/secondary	Resisted drug	*In vitro*/*vivo*	Core molecule	Alternative/combined drug	Reference
**2011**	786-O	Secondary	Sunitinib	*In vitro*	Lysosomal sequestration	–	(6)
**2012**	Caki-1	Secondary	Everolimus/VPA	*In vitro*	HDAC	VPA	(7)
**2013**	Caki-1	Secondary	VPA	*In vitro*, *vivo*	Akt	–	(8)
**2013**	786-O	Secondary	Sunitinib	*In vitro*	mTOR, PI3K	Rapamycin analogs	(9)
**2013**	786-O, ACHN	Secondary	Sunitinib/sorafenib	*In vitro*	β-Catenin Signaling	Ovatodiolide	(10)
**2013**	ACHN	Secondary	Sunitinib	*In vitro*, *vivo*	Akt	–	(11)
**2013**	ACHN, A498, Caki-1, Caki-2	Secondary	Vinblastine	*In vitro*	P-glycoprotein	–	(12)
**2013**	786-O	Secondary	Sunitinib	*In vitro*	EMMPRIN	–	(13)
**2013**	ACHN	Secondary	Temsirolimus	*In vitro*, *vivo*	mTORC2	–	(14)
**2013**	UMRC3	Secondary	Sunitinib	*In vivo*	Autotaxin	–	(15)
**2014**	786-O, A498, ACHN, CAKI1	Secondary	Sunitinib	*In vitro*	Sphingosine kinase-1	–	(16)
**2014**	Caki-1, KTCTL-26, A498	Secondary	Temsirolimus	*In vitro*	integrin α5, integrin β3	–	(17)
**2014**	Caki-1	Secondary	Temsirolimus	*In vitro*	cdk2, cyclin A	VPA	(18)
**2015**	Caki-1, KTC-26, A498	Secondary	Sunitinib	*In vitro*	cdk1, cdk2, Akt, Rictor, Raptor, p27	Everolimus	(19)
**2015**	786-O	Secondary	Sunitinib	*In vitro*	EZH2	–	(20)
**2015**	KURC1, KURC2, 786-O, Caki-1	Secondary	Sunitinib	*In vivo*	IL13RA2	–	(21)
**2015**	786-O	Secondary	Sunitinib	*In vitro*, *vivo*	PD-L1	–	(22)
**2015**	Caki-1	Secondary	Sunitinib	*In vitro*	EGFR	–	(23)
**2015**	Caki-1	Secondary	Sunitinib	*In vitro*, *vivo*	Reelin, Notch, BMP-6	–	(24)
**2015**	786-O	Secondary	Sunitinib, pazopanib, erlotinib, lapatinib	*In vitro*	Lysosomal sequestration	Everolimus	(25)
**2015**	786-O, RCC10	Secondary	Sunitinib	*In vitro*	Lysosomal sequestration	Elacridar, LLOM, bortezomib, MG132	(26)
**2016**	786-O	Secondary	Sunitinib	*In vitro*, *vivo*	AXL, MET	Cabozantinib	(27)
**2016**	ACHN	Secondary	Rapamycin	*In vitro*	GSK-3β, 4EBP1	–	(28)
**2016**	ACHN	Secondary	Sunitinib	*In vitro*, *vivo*	p44/42 MAPK, VEGFR-2	Axitinib	(29)
**2017**	769-P	Secondary	Sorafenib/sunitinib	*In vitro*	MUC13	–	(30)
**2017**	HK-2 KD, 786-O KD, Sor001	Primary	mTOR and tyrosine kinase inhibitors	*In vitro*, *vivo*	PDPK1	CYD-6-17	(31)
**2017**	ACHN, RCC23	Secondary	Sunitinib	*In vitro*	miR-575, miR-642b-3p, and miR- 4430 (↑), miR-18a-5p, miR-29b-1-5p, miR-431-3p, and miR-4521 (↓)	–	(32)
**2017**	786-O, ACHN	Secondary	Sunitinib	*In vitro*	CAV1	–	(33)
**2017**	786-O, UMRC2	Secondary	Sunitinib	*In vitro*	EZH2	–	(34)
**2018**	ACHN	Secondary	Sunitinib	*In vitro*	LAMP−2	–	(35)
**2018**	Caki-2	Secondary	Doxorubicin /vinblastine	*In vitro*, *vivo*	ABCC1	–	(36)
**2018**	786-O	Secondary	Metformin	*In vitro*	Histone H3 acetylation (↓)	VPA	(37)
**2018**	786O, ACHN	Secondary	Sunitinib	*In vitro*	RCAN1.4	–	(38)
**2019**	Caki-1	Secondary	Sunitinib	*In vitro*	miR-130b, PTEN	–	(39)
**2019**	786-O, OS-RC-2	Secondary	Sunitinib	*In vitro*	FZD1	–	(40)
**2019**	786-o	Secondary	Sunitinib	*In vitro*	miR-99a-3p, RRM2	–	(41)
**2019**	ACHN, Caki-1	Secondary	Sunitinib	*In vitro*	EMT-related genes	–	(42)
**2019**	786-O	Secondary	Everolimus	*In vitro*	miRNA-101, HIF-2α	–	(43)
**2019**	786-O	Secondary	Sunitinib	*In vitro*	COX-2, PGE2, CD133	–	(44)
**2019**	786-O, ACHN	Secondary	Sunitinib	*In vitro*, *vivo*	EIF3D, GRP78	–	(45)
**2020**	Caki-1, 786-O	Secondary	Sunitinib	*In vitro*, *vivo*	YB-1, ABCB-1	Elacridar	(46)
**2020**	786-O	Secondary	Pazopanib	*In vitro*, *vivo*	Gankyrin, STAT3, CCL24, CCR3	–	(47)
**2020**	786-O, ACHN	Secondary	Sorafenib	*In vitro*, *vivo*	miR-31-5p, MLH1	–	(48)
**2020**	786-O, A498, ACHN, caki1	Secondary	Sunitinib	*In vitro*, *vivo*	–	Rapalink-1	(49)
**2020**	786-O, A498, ACHN, Caki1	Secondary	Sunitinib	*In vitro*	RAB27B	–	(50)
**2020**	786-O, ACHN	Secondary	Sunitinib	*In vitro*, *vivo*	SNHG12, CDCA3	–	(51)
**2020**	786-O, OS-RC-2, TK-10	Secondary	Sunitinib	*In vitro*, *vivo*	CDK4-RB	Wogonin	(52)
**2020**	769P, 786O	Secondary	Sunitinib	*In vitro*	MALAT1, miR-362-3p, G3BP1	–	(53)
**2020**	786-O, ACHN	Secondary	Sunitinib	*In vitro*, *vivo*	YB1, EphA2	–	(54)
**2020**	ACHN, 786-O	Secondary	Sunitinib	*In vitro*, *vivo*	LINC00160, SAA1	–	(55)
**2020**	ACHN, 786-O	Secondary	Sunitinib	*In vitro*, *vivo*	CCAT1, c-Myc	–	(56)
**2020**	Caki-1,786-O, KTCTL26, A-498	Secondary	Sunitinib	*In vitro*	–	Artesunate	(57)
**2020**	Caki-1	Secondary	Sunitinib	*In vitro*	–	HDACI	(58)
**2020**	ACHN	Secondary	Sunitinib	*In vitro*	LAMP-2A, LAMP-2B	–	(59)
**2020**	ACHN, 786-O	Secondary	Sunitinib	*In vitro*, *vivo*	DAPK1	–	(60)
**2020**	786-O	Secondary	Sunitinib	*In vitro*, *vivo*	SLC1A5	–	(61)
**2021**	–	Secondary	VEGFR-TKIs	*In vitro*, *vivo*	ACE2, Ang- (1-7)	–	(62)
**2021**	786-O	Secondary	Sunitinib	*In vitro*, *vivo*	HIF-2, Plk1	Volasertib	(63)
**2021**	786-O	Secondary	Sunitinib	*In vitro*, *vivo*	TFE3	–	(64)
**2021**	786-O	Secondary	Sunitinib	*In vitro*	PI3K, AKT	G-1	(65)
**2021**	786-O, ACHN	Secondary	Sunitinib	*In vitro*	MX2	–	(66)
**2021**	–	Secondary	Sunitinib	*In vitro*	Lefty A	–	(67)
**2021**	786-O, A498, Caki-1	Secondary	Sunitinib	*In vitro*	miR-17~92 cluster, PD-L1	–	(68)
**2021**	Autochthonous Vhl/Trp53/Rb1 mutant ccRCC mouse model	Primary	PT2399	*In vivo*	Sphingosine-1-phosphate	FTY720	(69)
**2021**	786O	Secondary	Sunitinib	*In vitro*	PFKFB4	–	(70)
**2021**	HUVEC (human endothelial cell)	Secondary	Sunitinib	*In vitro*	–	Axitinib, sorafenib	(1)
**2021**	A498	Secondary	Imatinib	*In vitro*	PDIA6	–	(71)
**2021**	786-O	Secondary	Sunitinib	*In vivo*	BTRC, TRIM32	–	(72)
**2021**	786-O, ACHN	Secondary	Sunitinib	*In vitro*, *vivo*	circRNA_001895	–	(73)
**2021**	–	Secondary	Sunitinib	*In vitro*, *vivo*	circSNX6	–	(74)
**2022**	786-O	Secondary	Everolimus	*In vitro*	p-4EBP1, p-AKT, HIF1α, HIF2α	Norcantharidin	(75)
**2022**	Caki-1, SN12K1	Secondary	Sunitinib	*In vitro*, *vivo*	IL-6, VEGF, Bcl-2	Tocilizumab	(76)

↑ means high expression levels.

↓ means low expression levels.

### 1.2 Drug-Resistant Mechanisms Based on Traditional Models

Juengel et al. (7) used the Caki-1 RCC cell line to build secondary drug resistant models, and found that the resistance to everolimus owing to long-term mTOR suppression is prevented by valproic acid (VPA), a type of histone-deacetylase inhibitor *in vitro*. Caki-1 cells were treated twice a week, either with VPA (0.5 or 1 mM), everolimus (1 or 5 nM), or both for 2 versus 12 weeks, and a series of tests were performed with the two groups of cells. Cells cultivated with either drug alone for 12 weeks showed increased viability and decreased ratios of G0/G1 cells, indicating chronic resistance to either everolimus or VPA. However, combined drug administration increased the drug sensitivity of Caki-1 cells cultured for 12 weeks. The levels of H3 acetylation remained high, but Akt was not inactivated during combined drug administration, indicating the effects of histone H3 acetylation on the prevention of resistance to mTOR inhibitors in RCC. Juengel et al. (18) further discovered that everolimus resistance is associated with increased cdk2/cyclin A levels, promoting the transition of RCC cells into the G2/M phase. valproic acid (VPA) decreases the levels of cdk2/cyclin A, suggesting its potential as a treatment for patients with advanced RCC and acquired everolimus resistance. Su et al. (15) established an RCC xenograft mouse model with acquired resistance to sunitinib. Injections of UMRC3 cells were administered to BALB/c nude mice in both flanks, and the mice received sunitinib treatment (40 mg/kg) by oral gavage daily. The levels of ATX, an extracellular lysophospholipase D, were significantly upregulated in the endothelial cells of the sunitinib-resistant xenograft models, and combined application of Ki16425 (LPA1 antagonist) with sunitinib significantly increased the sensitivity of RCC to sunitinib, indicating that the acquired resistance to sunitinib is associated with aberrant activation of the ATX-LPA signaling pathway. Liu et al. (22) found that the tumor PD-L1 was upregulated after treatment of RCC cell lines and RCC xenografts in nude mice with sunitinib, suggesting that immunosuppression of the tumor microenvironment may induce resistance to antiangiogenic treatment in metastatic RCC. Zhou et al. (27) established an acquired sunitinib-resistant 786-O RCC cell line and xenograft mouse models to study the resistance mechanisms after chronic sunitinib treatment *in vivo* and *in vitro*. Time course assays and dose curves were performed to obtain a clear picture of the optimal treatment duration and dose to induce sunitinib-resistant 786-O cells, and 1 μM sunitinib for 2 weeks was chosen to induce sunitinib resistance. Activation of both AXL and MET, upregulation of EMT-associated genes such as Snail and β-catenin, and promotion of migrative and invasive abilities were found in acquired sunitinib-resistant 786-O RCC cells. Furthermore, angiogenesis was promoted in 786-O/HUVEC co-culture models pretreated with sunitinib. Two chronic sunitinib-treated xenograft mouse models were established for *in vivo* evaluations. For the first model, sunitinib-resistant and parental 786-O cells were injected into opposite flanks of NCr-nu/nu mice, fed for 9 weeks, and then sacrificed to harvest the tumor tissues for subsequent tests, which further indicated that chronic sunitinib pretreatment accelerates tumor proliferation and angiogenesis through the activation of AXL and MET. For the second model, 30 nude mice were subcutaneously injected with 1 × 10^7^ 786-O cells. After the tumors grew to 200 mm^3^, 20 mice were treated with sunitinib by oral gavage (20 mg·kg^−1^d^−1^) until the tumor growth progressed. Half of the sunitinib-resistant mice were switched to cabozantinib treatment (40 mg·kg^−1^d^−1^). Tumor growth was inhibited in the cabozantinib-treated group. Furthermore, AXL and MET were inhibited by cabozantinib, suggesting the ability of the drug to reverse acquired resistance to sunitinib in RCC.Zhou et al. (31) discovered a potent compound, CYD-6-17, that significantly inhibited the proliferation of drug-resistant RCC cells with different genetic profiles *in vitro* and exhibited *in vivo* efficacy in three established drug-resistant RCC cell lines: HK-2 KD and 786-O KD, which are resistant to mTOR and tyrosine kinase inhibitors, and Sor001, derived from sorafenib-resistant mRCC patients. 3-Phosphoinositide-dependent protein kinase 1 (PDPK1) is a potent AKT regulator and is associated with poor survival after targeted therapies. CYD-6-17 targets PDPK1 to kill drug-resistant RCC cells. Huang et al. (45) constructed 786-OR and ACHN-R cells with acquired sunitinib resistance and found that EIF3D is overexpressed in 786-OR and ACHN-R cells compared with that in 786-O and ACHN cells, which is consistent with EIF3D levels being upregulated in sunitinib-resistant RCC tissues compared with chemosensitive RCC tissues. Mechanistically, EIF3D enhances GRP78 stability by blocking ubiquitin-mediated proteasomal degradation of GRP78. 786-OR cells with or without LvshEIF3D and/or GRP78 were also subcutaneously injected into nude mice who were either administered sunitinib or a control treatment. The results show that EIF3D inhibition increases the sensitivity of RCC tumors to sunitinib, which could be reversed by GRP78 treatment.D’Costa et al. (46) found that YB-1 and ABCB-1 are overexpressed in sunitinib-resistant RCC samples compared to sensitive samples. They further constructed acquired sunitinib-resistant RCC cell lines and xenograft mouse models with Caki-1 and 786-O, and found that ABCB-1 inhibition with elacridar, combined with sunitinib, reverses sunitinib resistance development *in vitro* and *in vivo*. Wang et al. (47) demonstrated that pazopanib resistance in ccRCC results from activation of the autocrine regulatory loop of Gankyrin/STAT3/CCL24/CCR3 with acquired pazopanib-resistant of RCC cell line 786-O and xenograft mouse models. Liu et al. (51) established two sunitinib-resistant RCC cell lines, 786-O-R and ACHNR, and found that SNHG12 and CDCA3 levels are higher in sunitinib-resistant cells. Mechanistically, SNHG12 promotes CDCA3 transcription by increasing the stability of SP1. The authors then subcutaneously injected ACHN-R cells into nude mice to establish a xenograft model and demonstrated that tumor growth and sunitinib resistance could be reversed through SNHG12 (a long noncoding RNA) inhibition *in vivo*, concluding that SNHG12-regulated CDCA3 may be one of the numerous sunitinib-resistant mechanisms in RCC. Gotink et al. (6) found lysosomal sequestration as an important mechanism of sunitinib resistance using the 786-O cells that were acquired resistant to sunitinib. The concentration of sunitinib in resistant cells was 1.7- to 2.5-fold higher than that in untreated parental cells because of increased intracellular sunitinib distribution to acidic lysosomes. But the levels of p-Akt and p-ERK 1/2 were comparable between the two groups, indicating lysosomal sequestration reduced effectiveness of sunitinib. They further found that sunitinib-resistant 786-O cells were cross-resistant to pazopanib, erlotinib and lapatinib (25). Cross-resistance to TKIs was also observed in pazopanib- and erlotinib- resistant 786-O cells, which includes increased intracellular drug accumulation accompanied by increased lysosomal storage (25). Zhitomirsky et al. (77) also revealed lysosomal sequestration of hydrophobic weak base chemotherapeutics could trigger multidrug resistance of malignancies. Giuliano et al. (26) further constructed acquired sunitinib-resistant RCC cell lines (786-OR and RCC10R) and found lysosomotropic drugs or proteasome inhibitors could reverse sunitinib resistance *in vitro*. Mechanically, sunitinib stimulated the expression of ABCB1, an ATP binding cassette (ABC) transporter that promotes the accumulation of sunitinib in autolysosomes. The sunitinib-resistant cells could be resensitized through inhibition of ABCB1 by elacridar or increasing the permeability of lysosome membranes by Leu-Leu-O-methyl (LLOM).

## 2 Patient-Derived Drug Resistance Models

Recently, patient-derived RCC cell lines or xenograft mouse models have been increasingly applied in drug resistance research of RCC. Primary cultures of RCC tissue samples from patients with drug resistance can be used to establish new drug-resistant cell lines. Drug-resistant tumors could also be directly xenografted into nude mice to construct *in vivo* drug resistance models. In addition, the drug-resistant samples from RCC patients can be directly tested to detect the *in vivo* expression levels of target genes. **Table 2** lists the studies on RCC drug resistance using patient-derived *in vitro* and *in vivo* models. Primary RCC tumors and metastases can be collected during surgery or biopsy. Pleural or ascitic effusions and plasma of patients may also be used for subsequent research.

**Table 2:** Studies on RCC drug resistance using patient-derived *in vitro* and *in vivo* models.

Year	Model	Primary/secondary	Resisted drug	*In vitro*/*vivo*	Core molecule	Combined drug	Reference
**2013**	CcRCC tissues	Primary	sunitinib	*In vivo*	microRNA-141	–	(78)
**2013**	Papillary RCC cells from ascitic fluid	secondary	PF-04217903	*In vivo*	MET kinase	–	(79)
**2013**	KMRM-S2	Primary	Sorafenib	*In vitro*	Angiogenesis related molecules	–	(80)
**2015**	RP-R-01, RP-R-02	Primary	Sunitinib	*In vivo*	EZH2	–	(20)
**2016**	CcRCC tissue	Secondary	Sunitinib	*In vivo*	mTOR	Everolimus	(81)
**2016**	Primary cultures of PDX	Primary	Sunitinib	*In vitro*, *vivo*	FGFR, ERK, unknown paracrine signalings	Dovitinib, PD173074	(82)
**2016**	Ren-01, Ren-02	Secondary	Sunitinib	*In vivo*	MEK1/2, ERK1/2, MDSC	PD-0325901	(83)
**2017**	TKI-resistant patients	Primary, secondary	Sunitinib/sorafenib	*In vivo*	GLUT-1	Everolimus	(84)
**2017**	RCC tissues	primary	sorafenib	*In vivo*	lncRNA-SRLR, IL-6, STAT3	–	(85)
**2017**	PDC, PDX	Primary	TKIs, everolimus	*In vitro*, *vivo*	–	–	(86)
**2017**	RP-R-01, RP-R-02, RP-R-02LM	Primary	Sunitinib	*In vivo*	EZH2	–	(34)
**2018**	Patients	–	Sunitinib, Axitinib	Clinical trial	–	–	(87)
**2018**	RCC tissues	Primary	Sunitinib/pazopanib/sorafenib	*In vivo*	miR-9-5p	–	(88)
**2018**	Plasma of RCC patients	Secondary	Sunitinib	*In vivo*	S1P	–	(89)
**2018**	RCC tissues of patients	Secondary	Everolimus/temsirolimus	*In vivo*	PBRM1	–	(90)
**2018**	CcRCC tissues of patients	Primary	Sunitinib	*In vivo*	BCRP/ABCG2	–	(91)
**2019**	CcRCC tissues of patients	Primary/secondary	TKI	*In vivo*	EMT-related genes	–	(42)
**2019**	RCC tissues of patients	Primary	Sunitinib	*In vivo*	QPCT	–	(92)
**2019**	RCC tissues of patients	Primary	TKIs	*In vivo*	Adiponectin, AdipoR1	–	(93)
**2019**	RCC tissues of patients	Primary	Sunitinib	*In vivo*	EIF3D, GRP78	–	(45)
**2019**	Patients	Primary	PD-1/PD-L1 inhibitors	Clinical trial	–	–	(94)
**2020**	RCC tissues of patients	Secondary	TKIs	*In vivo*	TNFR1	–	(95)
**2020**	RCC tissues of patients	Primary	Sunitinib	*In vivo*	YB-1, ABCB-1	elacridar	(46)
**2020**	RCC tissues of patients	Secondary	PT2385	*In vivo*	HIF-2	–	(96)
**2020**	RCC tissues of patients	Primary	Sunitinib, Sorafenib	*In vivo*	PTEN	–	(97)
**2020**	Patients	Primary	TKIs and/or ICIs	Clinical trial	–	lenvatinib plus everolimus	(98)
**2020**	RCC tissues of patients	Primary	Sunitinib	*In vivo*	MALAT1, miR-362-3p, G3BP1	–	(53)
**2020**	fecal samples	–	ICBs	*In vivo*	Antibiotics	–	(99)
**2020**	Blood samples of patients	Primary	Pazopanib	*In vivo*	SDF-1, VEGF-A	–	(100)
**2020**	RCC tissues of patients	Primary	Sunitinib	*In vivo*	CCAT1, c-Myc	–	(56)
**2020**	RCC tissues of patients	Primary	Sunitinib	*In vivo*	LAMP-2A, LAMP-2B	–	(59)
**2020**	RCC tissues of patients	Primary	PD-1 inhibitors	*In vivo*	CD8+ T cell	–	(101)
**2021**	RCC tissues of patients	Primary	PD-1 inhibitors	*In vivo*	AXL, PD-L1	–	(102)
**2021**	RCC tissues of patients	Primary	Sunitinib	*In vivo*	HIF-2, Plk1	Volasertib	(63)
**2021**	RCC tissues of patients	Primary	ICIs	*In vivo*	TDO	–	(103)
**2021**	RCC tissue and plasma of patients	Primary	Sunitinib	*In vivo*	CTCF, QPCT	–	(104)
**2021**	RCC tissues of patients	Primary	Sunitinib	*In vivo*	P53	–	(105)
**2021**	RCC tissues of patients	Primary	Sunitinib	*In vivo*	CD44	–	(106)
**2021**	RCC tissues of patients	–	Nivolumab	*In vivo*	TCR, GZMB/K, HERV	–	(107)
**2021**	RCC tissues of patients	Primary	Imatinib	*In vivo*	PDIA6	–	(71)
**2021**	RCC tissues of patients	Primary	Sunitinib	*In vivo*	circRNA_001895	–	(73)
**2021**	RCC tissues of patients	Primary	Sunitinib	*In vivo*	CircSNX6	–	(74)
**2021**	Tri-culture model (cancer cells, endothelial cells, and patient-derived immune cells)	Primary	Cabozantinib	*In vitro*	CD4+ T cells	–	(108)
**2022**	RCC tissues of patients	Secondary	Sunitinib	*In vivo*	Cancer-associated fibroblasts	–	(109)
**2022**	Human fibroblasts from skin biopsies of a normal individual	Secondary	Sunitinib	*In vitro*	Cancer-associated fibroblasts	–	(109)

### 2.1 Primary Culture Models

Primary culture models can be established using RCC tissues from drug-resistant patients through a series of processes including tumor resection, tissue digestion, cell separation and purification, and primary cell culture. The drug-resistant primary RCC cell lines better represent and mimic the physiological and pathological characteristics of the individuals, providing better models for researches on either mechanisms or individualized treatments.

Karashima et al. (80) established a primary sorafenib-resistant ccRCC cell line, KMRM-S2, from a cutaneous metastasis of a 62-year-old man with metastatic RCC of the scalp. The metastatic RCC tissue of the cephalic skin was resected, washed with saline, cut up with sterile scalpels, and digested with serum-free solution containing collagenase type I and deoxyribonuclease I. Undigested tissue debris was removed through a gauze filter, and the cells were washed and incubated in medium containing 10% fetal bovine serum, insulin, human recombinant epidermal growth factor, GA-100, hydrocortisone, T3, epinephrine, and transferrin at 37°C. Thus, the ccRCC cell line KMRM-S2 was established. KMRM-S2 was confirmed to have a higher resistance to sorafenib *in vitro*. Cytogenetic abnormalities, such as hypertriploidy and translocation, were also observed in KMRM-S2.

### 2.2 Patient-Derived Xenograft Models

Patient-derived xenografts (PDXs) are tumors from patients directly implanted in nude mice, which stably preserve the molecular signature of patient tumors, including DNA copy number alterations, gene expression levels, and mutations, which are more physiological than conventional tumor cell lines (110). The transplantations can be either subcutaneous or orthotopical. The tumors are more likely to form and the sizes are easier to be monitored in subcutaneous transplantation models. Orthotopic transplantation models provide a better original tumor microenvironment, however, the operations may be more complex (111).

Adelaiye et al. (20) established two sunitinib-resistant ccRCC models (RP-R-01 and RP-R-02) from two patients. RP-R-01 originated from a skin metastasis of a patient with sporadic ccRCC who was initially sensitive to sunitinib but developed resistance. The *VHL* gene was deleted from RP-R-01. RP-R-02 was obtained from a skin metastasis of a patient with hereditary ccRCC (VHL syndrome) who did not respond to sunitinib from the start. The two samples were cut into 1 mm^2^ pieces and implanted subcutaneously into nude mice for subsequent *in vivo* studies. Based on a dose-escalation schema (40-60-80 mg/kg of sunitinib) and a direct-increase schema (80 mg/kg of sunitinib), they observed that the tumor eventually developed resistance to sunitinib, but transient resistance could be overcome by dose increase. Jiménez-Valerio et al. (81) developed a patient-derived RCC xenograft mouse model based on the orthotopic implantation of primary biopsies from the tumors of ccRCC patients, in which acquired resistance to sunitinib was induced after chronic sunitinib treatment. All tumors were initially sensitive, but finally adapted to sunitinib antiangiogenic therapy. Metabolic symbiosis between the tumor cells distal and proximal to the surviving vessels was also observed in sunitinib-induced RCC acquired resistance. Metabolic symbiosis, a process regulated by the mTOR pathway, was found to be responsible for sunitinib resistance, which could be overcome by mTOR inhibitors such as everolimus (81). Tran et al. (82) established 27 primary cultures of PDXs. The xenografts were obtained from the kidney RCCs or RCC metastases of vein thrombus, brain, bone, adrenal gland, and pleural fluid. Five patients with RCC were resistant to sunitinib treatment. They tested the ability of primary RCC cultures to signal to endothelial cells (ECs) and fibroblasts in co-culture assays and to stimulate angiogenesis in chorioallantoic membrane assays. Primary RCC cultures supported EC survival and were further divided into sunitinib-sensitive and sunitinib-resistant groups. Thirteen were sensitive and fourteen (including five from sunitinib-resistant patients) were resistant. For the sunitinib-sensitive group, VEGFs, secreted by RCC cells, combined with VEGFRs on the membranes of Ecs, activated ERK to promote EC growth. This process can be inhibited by sunitinib, and sunitinib sensitivity is correlated with VEGR production. However, sunitinib-resistant RCC cells show less dependency on VEGF to promote EC survival. The combined application of sunitinib (VEGFR and PDGFR inhibitor) and dovitinib (VEGFR and FGFR inhibitor) inhibits ERK activity in Ecs, but only a small part of ERK in fibroblasts. They concluded that RCC could activate EC through VEGF-dependent and -independent pathways, and combined inhibition of VEGF/PDGF/FGF receptors could inhibit mitogenic signaling in Ecs but not in fibroblasts. The problem is that sunitinib does not directly inhibit the proliferation of RCC cells *in vitro* and *in vivo*, which seems counterintuitive. A possible explanation might be the acquired mutations and copy number alterations in the RCC cells that the author used in the experiments. Diaz-Montero et al. (83) established two RCC xenograft models from two patient-derived ccRCC cell lines, Ren-01 and Ren-02. Ren-02 was obtained from a subcutaneous metastasis of a 42-year-old man with metastatic ccRCC and disease progression after bevacizumab therapy, but the relevant information on Ren-01 is missing. Sunitinib was administered to the tumor-xenografted mice *via* oral gavage for 4 weeks at 40 mg·kg^−1^d^−1^ to acquire resistance. Phospho-MEK1/2, Phospho-ERK1/2, and accumulation of MDSCs were induced but could be reversed by switching from sunitinib to PD-0325901 or a combination therapy. Adelaiye et al. (34) established another PDX model, named RP-R-02LM, which is intrinsically resistant to sunitinib but not to the VEGF therapeutic antibody bevacizumab. They implanted RP-R-02 cells into the prostates of nude mice to select a metastatic population. Lung metastasis was found and reimplanted subcutaneously or orthotopically to harvest a pure metastatic population. The metastatic model, together with previous RP-R-01 and RP-R-02, was used to explore the effect of the histone methyltransferase EZH2 on sunitinib-resistant RCC *in vivo*. The acquired sunitinib-resistant RCC cell lines 786-O and UMRC2 were employed *in vitro*. The antiangiogenic and anti-metastatic effects of sunitinib were preserved, while its direct anti-tumor effects were lost because of kinome reprogramming, which inhibits the expression of proapoptotic and cell cycle regulatory genes. Downregulation of EZH2 suppresses RTK phosphorylation and resensitizes the cells to sunitinib. An interesting study by Derosa et al. (99) investigated the relationship between gut bacterial composition and resistance to immune checkpoint blockade (ICB) in RCC patients. Fecal samples from 69 patients with advanced RCC treated with nivolumab and 2994 healthy volunteers were collected. The results show that recent antibiotic use significantly reduces the objective response rates of ICBs and changes the composition of the fecal microbiota, facilitating the dominance of distinct species such as *Clostridium hathewayi*. To establish a cause-effect relationship between gut bacterial composition and ICB efficacy, RCC-bearing mice that received fecal transplant from RCC patients resistant to ICB were successfully compensated with fecal transplant from responding RCC patients, leading to the conclusion that gut bacteria composition, which is influenced by antibiotics, could impact the success of ICB therapy in RCC patients.

Humanized mice with the immune system are newly developed animal models. The hematopoiesis of immunodeficient mice is destroyed by radiation of the bone marrow. Then Hematopoietic stem cells (HSC) derived from human are injected into the tail vein or bone marrow cavity to reestablish a human immune system. Hu-PDX (humanized patient-derived xenograft) model, which is established after transplantations of human tumor tissues into humanized mice with the immune system, can better simulate the interactions of human tumors, tumor microenvironment and the human immune system (112–114). The HU-PDX model shows great advantages in cancer immunotherapy research and has been used to research colorectal cancer, liver cancer, and triple-negative breast cancer. However, this new model has not been established in the immunotherapy research of RCC, which may to be developed in the future.

### 2.3 Other Patient-Derived Models

However, due to the complicated procedures to establish primary cultures and PDXs, most clinical drug-resistant samples from RCC patients were used for investigations of gene expression levels simply with some quantitative or semi-quantitative techniques like qPCR, western blot, or immunohistochemistry. Generally, the genes that are highly expressed in drug-resistant RCC samples are more likely to function as drug resistance genes, while those that are suppressed in drug-resistant RCC samples may resist drug resistance. Immunotherapy is an important treatment method for advanced RCC. Traditional cytokines like ILs and interferons show very limited therapeutic effects. Recently, cytokine-induced killer cells, VEGF monoclonal antibodies like Bevacizumab, and immune checkpoint inhibitors like PD1/PDL1 inhibitors have been widely used in the treatment of advanced RCC. Despite the promising therapeutic effects, immune responses may be gradually attenuated by varies of adaptive mechanisms. Due to the difficulties of establishing models resistant to varies of immunotherapies, direct examinations of the tumor specimens from RCC patients may be an easy and effective way for investigations of resistance mechanisms.

Berkers et al. (78) found that resistance to the multitargeted receptor tyrosine kinase inhibitor sunitinib in metastatic clear cell RCC (ccRCC) is associated with miR-141 downregulation-induced hypoxia resistance and epithelial-to-mesenchymal transition *in vitro* and *in vivo*. In this study, 20 freshly frozen metastatic ccRCC tissue specimens were included according to the following criteria. A) Patients underwent both first-line sunitinib therapy (50 mg/d, 4 weeks on/2 weeks off) and subsequent cytoreductive nephrectomy. B) Postoperative pathology confirmed the diagnosis of ccRCC. C) There should be at least one synchronous metastasis. Nine patients with progressive disease within six months were included in the poor response group, and eleven patients with at least one-year progression-free survival were included in the good response group. The expression of miR-141 decreased with epithelial-to-mesenchymal transition promoted in the tumors of the poor response group compared with those of the good response group, as determined by immunohistochemistry and *in situ* hybridization. A series of *in vitro* tests confirmed that miR-141 suppresses epithelial-to-mesenchymal transition and cell proliferation under hypoxic conditions. Diamond et al. (79) reported a patient with progressive papillary RCC with a heterozygous MET mutation at M1268T. Palliative debulking surgery was performed because of disease progression after two months of sequential treatment with sunitinib, temsirolimus, and ENMD-2076 (aurora and angiogenic kinase inhibitor), and the tumor tissue was obtained during surgery. The heterozygous MET mutation at M1268T results in a methionine-to-threonine change in the MET kinase domain, leading to abnormal activation of MET phosphorylation (115, 116). Thus, PF-04217903, a small-molecule MET inhibitor, was administered to the patient. Tumor size decreased by 35% after 53 weeks. The patient was asymptomatic for 26 months during PF-04217903 treatment, but finally succumbed to rapid tumor progression. Malignant cells from ascitic fluid were collected and conserved. The tumor specimens before and after PF-04217903 treatment were used for subsequent *in vivo* tests. They found that tandem duplication of the mutated MET allele occurred in almost 50% of the tumor cells, indicating that the acquired resistance to MET inhibitor PF-04217903 might be related to an increase in the copy number of the gene with the mutated MET allele. A total of 161 RCC patients receiving surgical resections were divided into sorafenib-treated (51 patients), nonsorafenib-treated (44 patients), and another independent group (66 patients) to evaluate PFS and response to sorafenib (85). The upregulation of a sorafenib resistance-associated lncRNA (lncRNA-SRLR) was identified in intrinsically sorafenib-resistant human RCC tissues. LncRNA-SRLR induces the IL-6/STAT3 axis to induce sorafenib resistance in RCC, and lncRNA-SRLR inhibition sensitizes nonresponsive RCC cells to sorafenib treatment *in vitro*. Xu et al. (89) compared the S1P levels in plasma from a small group of ccRCC patients (n = 20) who received sunitinib treatment as first-line therapy. The average S1P concentration was significantly increased when sunitinib resistance was established compared to when sunitinib treatment was performed (31.19 ± 10.13 nmol/ml vs. 22.89 ± 7.17 nmol/ml). Sphk1 promotes the proliferation and migration of RCC *via* activation of the AKT/mTOR pathway. Subsequent experiments confirmed that Sphk1 suppression increases the sensitivity of RCC to sunitinib *in vitro* and *in vivo*. Zhao et al. (92) used four pairs of sunitinib-responsive and resistant patient RCC tissues to analyze the methylation-differentiated CpG sites between the two groups using an Illumina Human Methylation 850 K microarray; a significant reduction in methylation degree was found in the QPCT promoter region of the sunitinib-nonresponsive tumors. Immunohistochemical assays, qRT-PCR, and western blotting confirmed the upregulation of QPCT expression in sunitinib-nonresponsive tumor tissues. Mechanistically, QPCT increased the stability of HRAS in inducing sunitinib resistance in RCC *in vitro*. PT2385 is a first-in-class HIF-2 inhibitor that treats ccRCC by dissociating HIF-2 complexes and inhibiting HIF-associated target gene expression. A prospective clinical study (96) involved patients undergoing treatment with this new drug. The acquired resistance to PT2385 was induced by prolonged treatment, and a gatekeeper (G323E) mutation was recognized in the acquired PT2385-resistant RCC tissues of patients. Mechanistically, the G323E substitution prevents HIF-2 dissociation by PT2385 to induce PT2385 resistance in renal metastasis. Braun et al. (101) analyzed 592 tumor samples from patients with advanced ccRCC treated with PD-1 inhibitors by whole-exome and RNA sequencing, integrated with immunofluorescence analysis. They found that the tumors resistant to PD-1 blockade were highly infiltrated with CD8+ T cells, only 27% had a non-infiltrated phenotype. Favorable PBRM1 mutations were depleted and unfavorable chromosomal losses of 9p21.3 were enriched in the infiltrated tumors compared with non-infiltrated tumors, suggesting the therapeutic response may be impacted by the potential interplay of immunophenotypes with somatic alterations. Terry et al. (102) examined 316 ccRCC samples from the advanced patients receiving the PD-1 inhibitor nivolumab after failure of antiangiogenic therapy. They found that AXL expression was strongly associated with PD-L1 expression and the RCC tumors with high levels of AXL and PD-L1 were resistant to PD-1 blockade.

## 3 Three-Dimensional Culture Drug Resistance Models

### 3.1 Advantages and Disadvantages of 3D Culture Models

Three-dimensional culture is a novel cell culture method that uses a variety of biochemical and tissue engineering techniques to grow cells into aggregates or spheroids rather than monolayers that adhere to the wall. Compared to traditional 2D culture, 3D culture better mimics *in vivo* cell growth patterns, providing a more physiological approach. The 3D spheroids present both metabolic and proliferative gradients influenced by the gradients of blood, oxygen, and nutrient supply, as well as the gradient of pH and metabolic-production accumulation across their geometry (117). The blood, oxygen, and nutrient supplies gradually decreased, with an increase in acidic metabolic products from the spherical surface to the center. Spherical tumors of approximately 500 µm may undergo central necrosis, with a viable layer of 200 µm surrounding the outer surface of the necrotic core (118, 119). Cells near the center adapt to the hypoxic microenvironment and exhibit decreased metabolism and proliferation, whereas the well-supplied outer layer cells grow faster (120). Changes in nutrient availability and cell contacts may alter the expression of genes associated with metabolism, proliferation, migration, invasion, differentiation, and communication (121–123). In addition, the drug concentration inside the spheroids may be affected by their different permeation capacities. The gene expression patterns of 3D cultured tumor spheroids are more similar to those in native tissues or primary tumor samples (117, 124, 125). Because intercellular communication and hypoxia are important factors influencing the therapeutic effects, toxicities, and resistance of drugs (126–129), 3D culture models seem to be a better choice for the investigation of drug resistance mechanisms.

However, the relatively high costs, difficulties in generating standard and uniform spheroids, and difficulties in developing co-culture models combining tumor cells with endothelial cells or cancer-associated fibroblasts, have limited the widespread application of 3D culture (121, 130, 131). 3D models cannot substitute *in vivo* models because complex *in vivo* tumor microenvironments are not completely recapitulated by 3D culture (117). Different types of cells, such as ECs, fibroblasts, and immune cells, may influence tumor genesis and development *via* communication with tumor cells. EC-induced vasculature can promote tumor growth and metastasis *in vivo* by providing nutrient and oxygen supply and increasing tumor adaptation and invasiveness (132–135). Three-dimensional co-cultures combining tumor cells with stromal cells have been developed to better mimic the *in vivo* microenvironment.

### 3.2 Establishment Methods of 3D Culture Models

There have been a variety of 3D-spheroid culture techniques, as listed below.

#### (1) Hydrogels

Hydrogels are natural or synthetic polymers that possess elevated water content. Natural hydrogels are originated from extra cellular matrix (ECM) and contain large amounts of endogenous bioactive molecules for cell growth and metabolism. But sometimes the unwanted or unknown components from natural hydrogels might be interference factors for mechanism researches. Synthetic hydrogels can overcome this defect due to their simple and specific components, but the cell function within synthetic hydrogels may be influenced perhaps due to lack of some beneficial endogenous components (136).

#### (2) Cell-Aggregating Methods

- Hanging drop method This method utilizes a specially designed culture plate with a small hole at the bottom of each well. The geometrical structure can guide cells along with culture medium to pass through the hole and form a stable droplet. The method can produce spheroids with a similar size, but the culture medium cannot be replaced.
- Ultra-low attachment method This scaffold-free method utilizes agarose or other specially synthesized materials to prevent cell attachment to the surface, promoting spontaneous aggregation of the suspended cells (137). an Ultra low attachment 96-well round bottom plate from Corning were used in a 3D culture monitoring tumor growth (138). Untreated polystryrene is hydrophobic and neutral. Cell adhesion proteins cannot properly adhere to this surface, and cell growth on this surface is asymmetrical and not well. Tissue-culture (TC) treated polystryrene presents a negative, hydrophilic surface. Cell adhesion proteins can properly adhere to this surface, providing a good condition for cell adhesion and growth. The ultra low attachment surface is a neutral, hydrophilic hydrogel covering. This surface minimizes the adherence and extension of cells by greatly inhibiting the adhesion of adhesion proteins.
- Magnetic levitation Cells are incubated with magnetic nanoparticles to acquire magnetism, then suspended and aggregated at the air-liquid interface of the culture medium by a magnetic field (117).
- Non-stagnant methods Non-stagnant methods use physical means like shaking or rotation to prevent cell adhesion to the surface of culture dishes. Cells lacking a substrate will spontaneously aggregate to establish intercellular communications.

### 3.3 Drug-Resistant Mechanisms Based on 3D Culture Models

Recently, 3D culture has been reported as a means of investigating RCC drug resistance. Brodaczewska et al. (139) established 3D spheroid RCC models. The RCC cell lines Caki-1 and ACHN were cultured in serum/growth-factor-deprived medium on laminin-coated or poly D-lysine-coated plates. Small spheroids of ACHN cells and large spheroids with Caki-1 cells exhibiting reduced central cell viability were formed in the ECMs. The expression levels of stem cell markers (CD105 and CD133) and stem cell transcription factors (OCT4, SOX2, and NES) were higher in 3D spheroids than in adherent 2D cultures. In addition, epirubicin, sunitinib, and doxycycline presented better sensitivities in RCC cell monolayers than 3D spheroids. The results demonstrate that 3D-cultured RCC cells present a stem−like phenotype and stronger drug resistance than traditional 2D-cultured cells.

As previously mentioned, 3D models can better mimic both low-oxygen-tension-related pathways and cell-cell dynamics in tumor-like spatial structures. To investigate the relationship between hypoxia and TKI resistance in RCC, Bielecka et al. (140) established hypoxic 3D *in vitro* RCC models with human papillary kidney cancer stem-like cells (HKCSCs) in soft agar and suspension culture. Hypoxic HKCSCs have an increased ratio of quiescent cells. Hypoxia also induces map2k1 overexpression and sorafenib resistance in pRCC. 3D spheroid cultures of patient-derived tumors are useful for investigating the mechanisms of tumor stemness because this technique promotes the increase of tumor cells with stemness properties (141–143). Dipeptidyl peptidase IV (DPP4) has recently been regarded as a new tumor stemness-related protein. Kamada et al. (144) established patient-derived RCC spheroids and found a positive correlation between DPP4 and other stemness-related genes. DPP4 inhibition reverses the sunitinib resistance of RCC *in vitro* and *in vivo*. Rausch et al. (145, 146) established a 3D heterotypic spheroid co-culture model to assess the therapeutic effect of an optimized low-dose synergistic drug combination (ODC) consisting of four tyrosine kinase inhibitors, namely osimertinib, pictilisib, AZD4547, and AZD8055. Scaffold-free heterotypic spheroidal cultures of 700 sunitinib-resistant Caki-1 cells, 200 human fibroblasts NHDFα, and 100 human endothelial cells ECRF24 were prepared in 96-well low-attachment U-bottom plates to mimic the physiological characteristics of ccRCC. The cell metabolic activity in the 3D heterotypic co-cultures decreased by >80%, remaining inactive in non-cancerous cells after the ODC treatment, showing the efficacy of this low-dose combination in sunitinib-resistant RCC.

## 4 Transgenic Drug Resistance Models

### 4.1 Establishment Methods of Transgenic Drug Resistance Models

The drug resistance of RCC is usually related to the abnormal expression of related signal pathways. Intervening the expression of key molecules of related pathways through transgenic technology to affect the drug sensitivity of RCC has become an important tool to study its drug resistance mechanism. Clustered regularly interspaced short palindromic repeats (CRISPR)-associated protein 9 (Cas9) is an RNA-guided DNA endonuclease derived from the type II CRISPR bacterial immune system. CRISPR/Cas9 has been widely used as an efficient gene-editing system based on the ability to target new genes simply by altering the sequences of single guide RNAs (sgRNAs) (147, 148). The targeted-sequence specificity of Cas9 depends on both accurate Watson–Crick base pairing between its guide RNA and the target DNA site, and a direct interaction between Cas9 and a short protospacer adjacent motif (PAM) of DNA (149–153). The double-stranded DNA is catalytically cleaved by the two nuclease domains HNH and RuvC of Cas9 (152, 153). Mutations at the targeted sites may occur owing to a shift in the reading frame induced by random insertions or deletions (147). Homology-directed repair can also be achieved by homologous recombination with an introduced homologous donor DNA (154, 155).

### 4.2 Drug-Resistant Mechanisms Based on Transgenic Drug Resistance Models

Sunitinib, a multi-targeted receptor tyrosine kinases inhibitor, can prevent the progression of RCC by blocking VEGFR and PDGFR-β. However, the crosstalk between EGFR, PDGFR and VEGFR may induce drug resistance. Liu et al. used two gRNAs targeting exon 2 of EGFR with the homology-directed DNA repair (HDR) templates specific to the cut sites of EGFR, generating RC21 EGFR knockout cell line. They found that CRISPR-mediated ablation of overexpressed EGFR in combination with sunitinib could significantly improve the therapeutic effect of sunitinib. Interestingly, the loss of EGFR eventually induced resistance to SAHA and cisplatin (156). Several studies have also shown that the EGFR status is associated with drug resistance in cancer, which suggests that EGFR knockout RC21 cells could be a cisplatin-resistant cell model. To investigate the role of PTEN in TKI resistance to RCC, SEKINO et al. developed PTEN knockout cells in RCC cell lines using the CRISPR-Cas9 technique. They found that PTEN knockout promoted the spheroid formation and sunitinib resistance in RCC cells (97). Statistical analysis showed a significant association of negative PTEN expression with poor PFS in metastatic RCC treated with sunitinib and sorafenib or sunitinib alone. The PTEN knockout RCC cell lines may be a sunitinib-resistant cell model. Makhov et al. (157) used CRISPR/Cas9-based high-throughput loss of function (LOF) screening to identify sunitinib-resistant cell factors. The procedures were described by Makhov et al. (157). Briefly, 786-O cells were transfected with lentiviruses carrying the *Cas9* gene, and successfully transfected cells were selected using puromycin. Then, 786-O cells were infected with lentiviruses carrying a human CRISPR sgRNA library targeting 18,000 genes with 90,000 individual sgRNAs (5 sgRNAs per gene) with MOIs < 1 and selected by blasticidin. Multiple genes were knocked out after doxycycline-induced *Cas9* expression. Some of the knockout 786-O cells were further cultured with 10 μM sunitinib for 12 days (approximately six passages). The 786-O cells without *Cas9* induction and the knockout 786-O cells with or without sunitinib were cultured and conserved for chromosomal DNA purification and deep sequencing of sgRNAs to identify the sgRNAs underrepresented in the surviving cell population. Cells with essential genes knocked out would be eliminated after induction of Cas9 expression, whereas the cells with their non-essential genes knocked out would remain. Likewise, cells with knocked out genes that contribute to sunitinib resistance, should be eliminated from the population when incubated with sunitinib. Based on this, a large number of sunitinib-resistant genes were identified with farnesyltransferase (FTase) among the top hits. Subsequent *in vitro* and *in vivo* experiments demonstrated that inhibition of FTase with lonafarnib significantly increases the anti-tumor efficacy of sunitinib. In conclusion, CRISPR/Cas9 LOF screening may be a promising method for the identification of genes involved in resistance to anti-tumor therapies.

## 5 Summary

**Figure 1:**
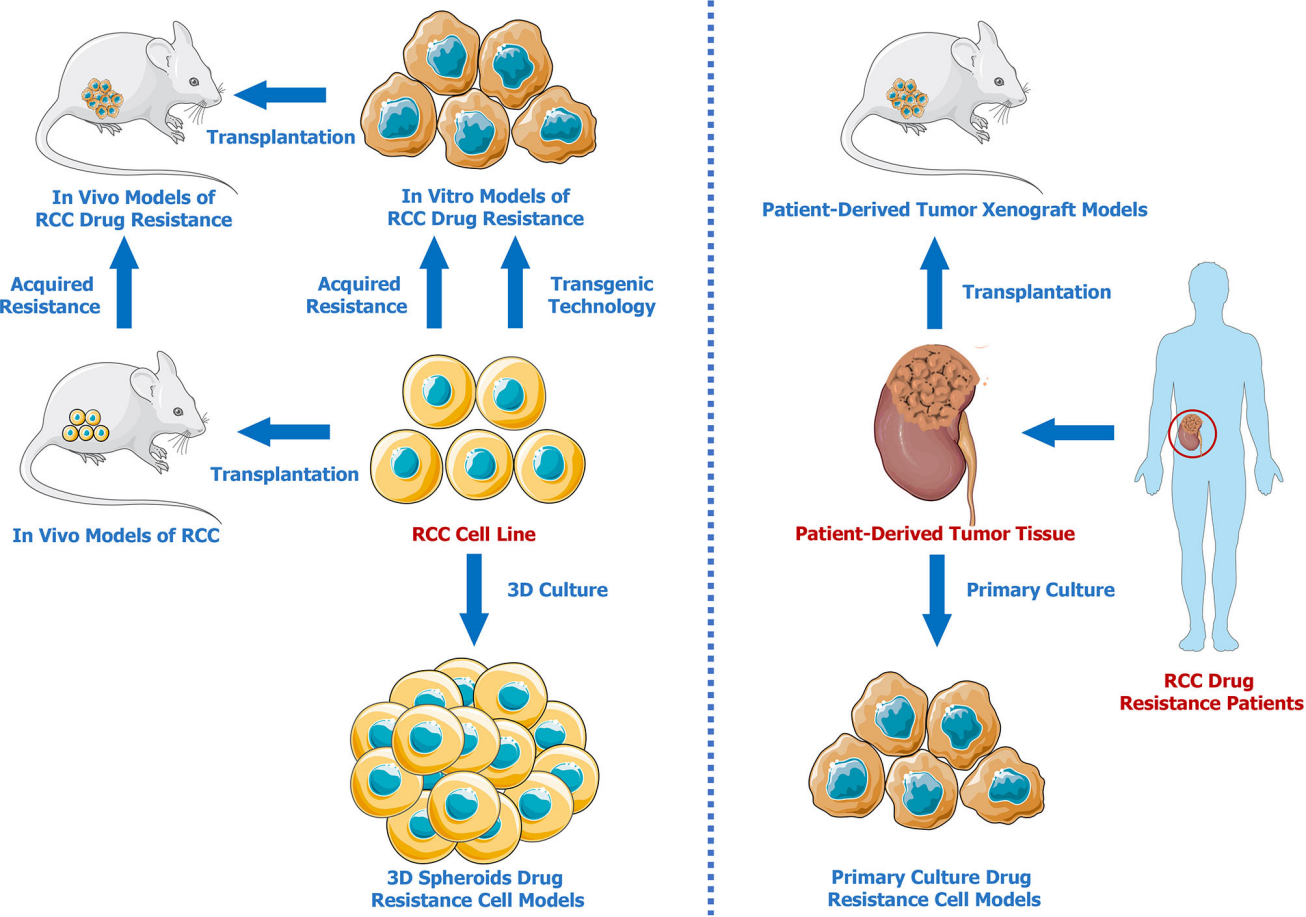
Renal Cell Carcinoma Drug Resistance Models.

Developing effective models is essential for investigating drug resistance mechanisms in RCC. Therefore, we summarized the advances in renal cell carcinoma drug resistance models (**Figure 1**). Compared to traditional commercial and mature RCC cell lines, patient-derived models present and retain better individual characteristics, which are vital for the investigation of the different drug resistance mechanisms with respect to different unique cancer pathological subtypes. Three-dimensional models better mimic the tumor microenvironment solely through simple and stable *in vitro* cultures, providing a better method for testing drug effectiveness and resistance. In addition, gene-editing techniques can be used to establish genetically modified cell lines or animal models that are resistant to specific drugs. It has to be admitted that none of the above mentioned methods are perfect. From our perspective, PDXs seem to be the optimal drug resistance models for researches on tumor mechanisms. There may be two reasons. First, patient-derived tumor xenografts preserve the molecular characteristics of patient tumors. Moreover, subcutaneous or orthotopical transplantations in animals provide more physiological *in-vivo* environments. It is expected that more PDX models and cutting-edge techniques will be applied in the future for further exploration of drug resistance mechanisms in RCC.

## Author Contributions

YX and HQ wrote the manuscript. HQ, GZ, JZ and JS provided the critical revisions. All authors approved the final version of the manuscript for submission and approved it for publication

## Funding

This work was supported by grants from Natural Science Foundation of China (82002809, 81902484), China Postdoctoral Science Foundation (2020M670864), Youth Support Project of Jilin Association for Science and Technology (202028), Medical and Health Talents Project of Jilin Province (2020SCZT097)

## Conflict of Interest

The authors declare that the research was conducted in the absence of any commercial or financial relationships that could be construed as a potential conflict of interest.

## Publisher’s Note

All claims expressed in this article are solely those of the authors and do not necessarily represent those of their affiliated organizations, or those of the publisher, the editors and the reviewers. Any product that may be evaluated in this article, or claim that may be made by its manufacturer, is not guaranteed or endorsed by the publisher.
